# Ecological Drift and Directional Community Change in an Isolated Mediterranean Forest Reserve—Larger Moth Species Under Higher Threat

**DOI:** 10.1093/jisesa/ieaa097

**Published:** 2020-09-19

**Authors:** Mirko Wölfling, Britta Uhl, Konrad Fiedler

**Affiliations:** Department of Botany and Biodiversity Research, University of Vienna, Rennweg, Vienna, Austria

**Keywords:** extinction risk, ecological specialization, functional dispersion, body size, long-term data

## Abstract

Long-term data are important to understand the changes in ecological communities over time but are quite rare for insects. We analyzed such changes using historic museum collections. For our study area, an isolated forest reserve in North-East Italy, data from the past 80 yr were available. We used records of 300 moth species to analyze whether extinction risk was linked to their body size or to their degree of ecological specialization. Specialization was scored 1) by classifying larval food affiliations, habitat preferences, and the northern distributional limit and 2) by analyzing functional dispersion (FDis) within species assemblages over time. Our results show that locally extinct species (mean wingspan: 37.0 mm) were larger than persistent (33.2 mm) or previously unrecorded ones (30.7 mm), leading to a smaller mean wingspan of the moth community over time. Some ecological filters appear to have selected against bigger species. By using coarse specialization categories, we did not observe any relationship with local extinction risk. However, FDis, calculated across 12 species traits, significantly decreased over time. We conclude that simple classification systems might fail in reflecting changes in community-wide specialization. Multivariate approaches such as FDis may provide deeper insight, as they reflect a variety of ecological niche dimensions. With the abandonment of extensive land use practices, natural succession seems to have shifted the moth community toward a preponderance of forest-affiliated species, leading to decreased FDis values. Multivariate analyses of species composition also confirmed that the moth community has significantly changed during the last 80 yr.

Ecological communities are dynamic networks that may respond to all kinds of environmental constraints (Vellend 2016). During the last decades, multiple anthropogenic stressors dramatically affected remaining near-natural systems, such as nature reserves, finally culminating in the definition of a new geological epoch: the Anthropocene (Laurance 2019). Landscapes are modified on large scale by anthropogenic land use, including agricultural intensification and urbanization (Plieninger et al. 2016). Besides the loss of suitable habitat area (Thompson et al. 2017), reduced connectivity between patches alters species immigration and genetic flux between isolated habitats (Habel and Schmitt 2018). Consequently, remnant patches of near-natural habitat areas are nowadays often isolated from one another and embedded in landscapes under intense human pressure.

The large-scale changes of terrestrial habitats have recently been shown to threaten regional insect diversity even inside conservation areas. Over the last 20 yr, scientists found a drastic decline in terrestrial insect biomass and gamma diversity (Seibold et al. 2019). Insects are the most species-rich group of terrestrial Eucaryota. They form an important part of manifold trophic interactions and ecosystem processes such as pollination, herbivory, and pest control (Noriega et al. 2018). Moreover, insects serve as a food resource for animals at higher trophic levels such as birds and bats (Maas et al. 2016). The landscape wide loss of insect species raises attention on how conservation management might be ameliorated to stop further biodiversity erosion and to maintain ecosystem function. Rösch et al. (2013) showed that insect species richness declined with increasing habitat isolation and especially with the reduced size of the remnant habitats. They observed highest extinction rates and lowest probabilities of recolonization in small, isolated fragments. Maintaining or restoring habitat connectivity therefore is an important goal in biodiversity conservation (Correa Ayram et al. 2016), especially as large remnants of near-natural habitats are particularly important for the maintenance of disturbance-sensitive and threatened taxa (Melo de Melo et al. 2019).

Besides the general observation of insect diversity decline within fragmented habitat patches, we lack in many cases information about species’ individual sensitivities to isolation and disturbance. Are there some species groups that face—due to their physiological or functional characteristics—a greater risk of local extinction inside isolated nature reserves? For vertebrates, numerous studies have shown that body size is an important factor shaping extinction risk (Ripple et al. 2017, Smith et al. 2018). Large-sized animal species are dependent on the primary productivity of their habitat, which plays an important role to nourish the individuals. Without sufficient food resources, no viable population can persist. Consequently, with decreasing habitat size, a lower carrying capacity for larger consumers is expected especially when additional stressors might reduce food quality. Indeed, also for insects, there is increasing evidence that larger species are under elevated extinction risk in isolated habitats (e.g., Koh et al. 2004, Nolte et al. 2019).

Looking at species functional traits, there is evidence for specialized species vanishing more strongly due to land use intensification (Mangels et al. 2017) and urbanization (Concepción et al. 2015). The phenomenon of specialized species being replaced by a few generalist ones is described as biotic homogenization of communities (Knop 2016). Fragmentation can also favor functional homogenization and the replacement of specialized species by generalist ones (Bagchi et al. 2018, Ramiadantsoa et al. 2018). Thus, Keinath et al. (2017) suggested that conservationists should pay particular attention to specialized species whenever anthropogenic disturbances could further fragment remaining habitats. However, there is no uniform method to measure the degree of specialization. For animals, a common approach is to classify species according to their feeding habits or habitat preferences. Either only one of these characteristics is considered (Mangels et al. 2017) or various specialization values covering different aspects of the ecological niche requirements of organisms are aggregated into one synthetic specialization score (Eskildsen et al. 2015). Additionally, different methods to quantify functional diversity have become popular at the community level. On the one hand, community weighted means can give insight into conditions favoring shifts with regard to single factors (Neff et al. 2019). On the other hand, functional dispersion (FDis) represents the abundance-based dispersion of species in trait space (Laliberté and Legendre 2010). As functional specialization is defined as the relative distance of a species from the centroid (Bellwood et al. 2006, Laliberté and Legendre 2010), FDis can be interpreted as mean functional specialization at the community level. To calculate FDis, a variety of ecologically relevant species traits needs to be assembled and subjected to multivariate analysis.

In this study, we evaluate if body size or functional specialization of insect species are linked to a higher risk of extinction in an isolated conservation area. To achieve that goal, we analyzed historic data (1933‒1996) and recent samples (1997‒2012) from the coastal pinewood reserve Pineta san Vitale (hereafter termed PsV) in North-East Italy, comprising a time span of about 80 yr. When no long-term monitoring data are available, museum collections can serve as important clues for community composition in the past. PsV, as most conservation areas in Europe, has become ever more disconnected from other remaining near-natural habitats over the past century through urbanization and the spread of intense agriculture (Andreatta 2010). So, the distances of PsV to other larger, near-natural areas are as follows: 32 km to Bosco Mesola (North), 40 km to the forests in the Apennines (west), and 10 km to Pineta di Classe (south). There is mainly agricultural land located between PsV and the other mentioned regions (Uhl et al. 2020).

Additionally, different potential pollution sources developed in the vicinity of the conservation area, like the industrial harbor of Ravenna (Airoldi et al. 2016), influencing the air (Lucialli et al. 2007) and water quality (Guerra et al. 2014) in the direct surroundings of the forest reserve. Finally, the vegetation structure inside the conservation area has changed due to the abandonment of former extensive management practices (wood and reed production, pine-nut harvest, cattle grazing) and the progress of natural forest succession (Wölfling et al. 2019). While developing toward a more natural forest habitat structure, early successional stages like open areas largely vanished. However, recent conservation management in the reserve tries to maintain different habitat structures, such as grassland and reed, by keeping horses inside the reserve area. Here, we explore multi-decadal changes in moth communities of PsV and investigate whether long-term isolation and habitat change had an effect on body size and functional specialization of the moth community. We address the following specific hypotheses:

1. Larger moth species have experienced a higher risk of local extinction in PsV, as the isolated area might fail to provide sufficient food resources and habitat quality. Subsequently, the mean wingspan of the whole community should have decreased over time.
2. Specialized species were more likely to go extinct, as they are more sensitive to environmental changes. The isolated forest remnant might be unsuitable to maintain specialist species and functional homogenization might have occurred inside the nature reserve. Therefore, the mean degree of specialization should have decreased over time.
3. Extinction and colonization events have contributed to directional shifts in the moth community composition in PsV over the last 80 yr. Taking into account the natural succession that has transformed the vegetation of PsV, a loss of open habitat species is expected.

## Materials and Methods

The forest reserve PsV (expansion of 950 ha from north 44°31′39.15″N, 12°14′19.82″E to south 44°27′48.09″N, 12°13′43.67″E and west 44°29′51.96″N, 12°13′22.79″E to east 44°29′50.50″N, 12°14′15.56″E) is located in the Emilia Romagna (Italy) close to the city of Ravenna. The area of PsV developed from the tenth century onwards through sedimentation forming dunes. The pine woods, which were planted afterward for firewood and pine-nut production in the 10th and 11th centuries, were also used extensively for cattle grazing keeping the understory open and therefore forming a very open forest structure (Malfitano 2002, Andreatta 2010). Extensive land use was then abandoned in 1988, when PsV became a part of the Parco Regionale del Delta del Po (Consorzio Del Parco Regionale Del Delta Del Po 2004) and therefore was protected as UNESCO biosphere reserve (UNESCO 2015). Since then, succession formed more forest like habitats with a diverse understory in PsV (Wölfling et al. 2019). Today, PsV consists mainly of downy oak forest and hygrophilous forest, but also smaller patches of pine stands, reed, and open habitats. PsV is protected under several levels of legislation based on Natura 2000 (Montanari 2010) and the Convention of Ramsar (Ramsar Convention Secretariat 2013). For the present paper, three historical moth collections were analyzed:

- Museo di Storia Naturale di Venezia (vouchers of 93 species from 1933 to 1968)
- Museo Civico delle Cappuccine, Bagnacavallo (vouchers of 41 species from 1966 to 1980)
- Private collection of Edgardo Bertaccini (vouchers of 157 species from 1977 to 1996)

Additionally, we sampled in PsV between 1997 and 2012. For each year, the samples should represent the reserve-wide species assemblage. Therefore, we always tried to sample the different habitats of PsV for each year and subsequently pooled the data into one species list for each year. Seven locations were sampled between 1997 and 2002 with a 500 W HWL manual light trap, mainly in early summer and early autumn in downy oak forest, riparian forest, reed, and open habitats. In 2011, we used weaker light tubes (15 W BL + 15 W white BL) in automated light traps at 20 locations to sample all mentioned vegetation types simultaneously in spring, early summer, high summer, and autumn to get the most complete dataset of the whole PsV moth community. The technical characteristics of the automated traps are different from that of the manual trap and can therefore cause a sampling bias (Axmacher and Fiedler 2004) that is discussed later. In 2012, again the 500 W HWL manual light trap was used like in the years 1997–2002 at nine different locations. In total, 237 species were sampled from 1997 to 2012. All vouchers from the own sampling are stored in the private collection of Mirko Wölfling (Niederwerrn, Germany).

Over a span of 80 yr, 300 moth species (Macroheterocera sensu Regier et al. 2013 plus Cossidae and Hepialidae) were considered for the subsequent analyses after phenological cleaning of all data. In particular, all early- and late-flying species as well as long-distance migrants and strictly diurnal species were removed, since these were not represented in our own light-trap samples. For the analysis, only incidence data were used, as we were not able to count abundances out of the historic collections. Further details about data sources and management were described in Wölfling et al. (2019).

For the first hypothesis, the typical wingspan (averages) of each species was extracted from a database (http://ukmoths.org.uk) or, if not available there, specimens were measured from our collections. The log-transformed wingspan served as a measure of body size.

For analyzing the hypothesis on overall specialization, we created a toolbox (Table 1) that considered three ecologically important dimensions for classifying all species according to their degree of specialization. The niche dimensions considered and their scoring are listed in Table 1. We chose descriptors pertaining to larval resource requirements, to the climatic niche, and the habitat use since these are essential aspects for defining how specialized a species is. Data on larval host selection were extracted from Ebert (1994‒2003) and from the websites www.euroleps.ch and www.pyrgus.de. The northern limit of the distribution of each species in Europe was taken from www.gbif.org. Specialization in habitat use (including their preference for open habitats) was extracted from Ebert (1994‒2003) and the two databases www.euroleps.ch and www.pyrgus.de. We scored each niche dimension on a rank scale from 1 (most specialized) to 4 (least specialized). Aggregating over these three dimensions, the degree of total specialization of each species is therefore the sum of the scores along all three dimensions. Accordingly, highly specialized species may attain a minimum value of 3 (3 × score 1) and highly generalist species may reach a maximum value of 12 (3 × score 4). A complete species list with all scorings regarding the different dimensions of specialization is provided in Supp Appendix 1 (online only).

**Table 1.** Register for scoring the specialization levels of each moth species

Dimension of resource	Classification factors	Classification value
Specialization in larval food selection	The species’ phagism-type is:	
	Monophagous within one plant genus	→ 1
	Oligophagous within one plant family	→ 2
	Polyphagous: >1 plant family	→ 3
	Highly polyphagous: >5 plant families	→ 4
Northern limit of distribution in Europe	The northern distribution limit of the species is:	
	43°–46° N (range limit: south of the Alps)	→ 1
	47°–50° N (range limit: German highlands)	→ 2
	51°–54° N (range limit: North or Baltic sea coast)	→ 3
	55°–71° N (range limit: further north than class 3)	→ 4
Specialization in habitat choice	The species occurs in:	
	1–2 habitat types (e.g., xeric grasslands, coppice forests, pine forests, etc.)	→ 1
	3–4 habitat types 5 or more habitat types or common/nonspecial habitat types (e.g., forest, deciduous mixed forest, edge of the woods, woody habitats, meadows, etc.)	→ 2 → 3
	Numerous and/or anthropogenic influenced habitats (gardens, parks, urban areas, common meadows	→ 4

Furthermore, we compiled a trait matrix containing information on 12 species traits of functional relevance, viz. wingspan, presence or absence of proboscis, larval food source (detritivore, lichen feeder, and 15 plant families), salt tolerance of larval food plants, use of ruderal food plants, growth form of larval host plants (e.g., woody or herbaceous), degree of larval food specialization (from monophagous to highly polyphagous), habitat type (forest, open habitats, shrub-land, or reed), phenology of adult activity period (spring, early summer or summer), voltinism, hibernating stage, and latitudinal extent of European distributional range. From these trait data, we calculated a Gower dissimilarity matrix, which was—together with the moth incidence data—used for calculating FDis as an index for community-wide specialization using the ‘dbFD’ function in the ‘FD’ package in R (Laliberté and Legendre 2010). Comparing these two different methods for classifying moth specialization, we wanted to analyze which index is more useful.

To analyze local extinction risk, we partitioned all moth species under consideration into three groups: species observed only before 1997 (named ‘lost’ hereafter), species recorded only after 1997 (named ‘previously unrecorded’), and species occurring before and after 1997 (named ‘persistent’). The year 1997 was chosen for separation because this was the year when our own sampling started. Body size was used as the response variable in a linear mixed-effect model with taxonomic family included as a random factor and species status as the fixed factor, using the R package ‘nlme’ (Pinheiro et al. 2019). Food specialization, northern limit of distribution, habitat specialization, and total specialization were used as response variables in separate generalized linear models with Poisson-type error distribution, again modeling species status as fixed and moth family as random factors.

Additionally, generalized additive models (hereafter GAM) were built to look for changes in body size and specialization over the observed time series. For the historic collection data, we had to pool some years since there were usually too few individuals present as vouchers in the collections per year. The historic museum collection data were split into four partitions, two for the Callegari/Martinasco collection (1933–1949 summed up as ‘1940s’ and 1950‒1976 summed up as ‘1960s’) and two more for the Bertaccini collection (1977‒1984 summed up as ‘1970s’ and 1985‒1996 summed up as ‘1980s’). Our own samples, which comprised far more vouchers, were split up by year. The calculation of the GAM was done using the ‘mgcv’ package (Wood 2011). The log-transformed mean wingspan of all observed moth species per year was used as the response variable. Furthermore, the annual degree of specialization at the community level was calculated as mean value of the specialization scores of all observed moth species per year. Similarly, annual FDis was calculated using the moth incidence data from each time period. The proportion of open habitat species (logit transformed) served as the response variable in GAM to analyze whether open habitat users may have decreased during the last decades due to succession.

Finally, to check whether changes in species composition in PsV over time were related to shifts in wingspan, ecological specialization, or preference for open habitats, we used non-metric multidimensional scaling (NMDS) and a permutation test (999 permutations). We first prepared our own sample data by separating them according to single sampling years, yielding eight data points. Museum collections data were as above split into the four partitions ‘1940s’, ‘1960s’, ‘1970s’, and ‘1980s’. From the resulting species × time layer incidence matrix, a triangular similarity matrix was calculated using the Sørensen similarity measure. As potential explanatory factors, we included community mean values of wingspan (log transformed), mean total specialization score, and the proportion of open habitat species (logit transformed) per each time unit. For visualization of possible temporal trends, we created an ordisurf diagram using the package ‘vegan’ (Oksanen et al. 2018). Ordisurf is a function performed on the NMDS ordination, based on GAM (Oksanen 2007).

## Results

**Fig. 1.**
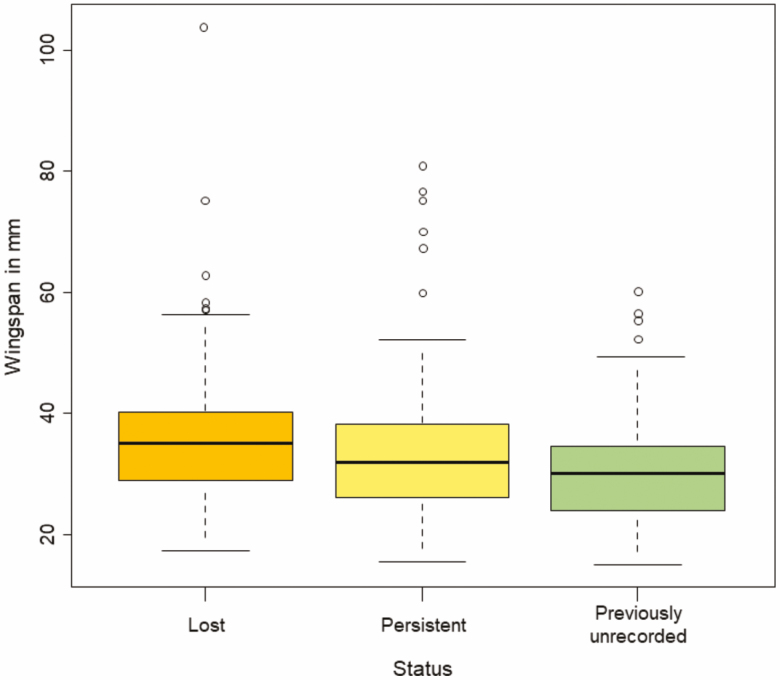
Wingspan of moth species from Pineta san Vitale (PsV) partitioned into three categories according to their occurrence status. ‘Lost’ (orange) represents moth species that were only found pre-1997 (*n* = 63). ‘Persistent’ (yellow) denotes moths occurring pre-1997 as well as afterward (*n* = 156). ‘Previously unrecorded’ (green) refers to species only observed in 1997 or later (*n* = 81). ‘Previously unrecorded’ and ‘Lost’ species differed significantly in body size (*P* = 0.002). Bar in the middle represent median, box limits are third and first quartiles, and whiskers describe data points within 1.5 times of the interquartile range.

**Fig. 2.**
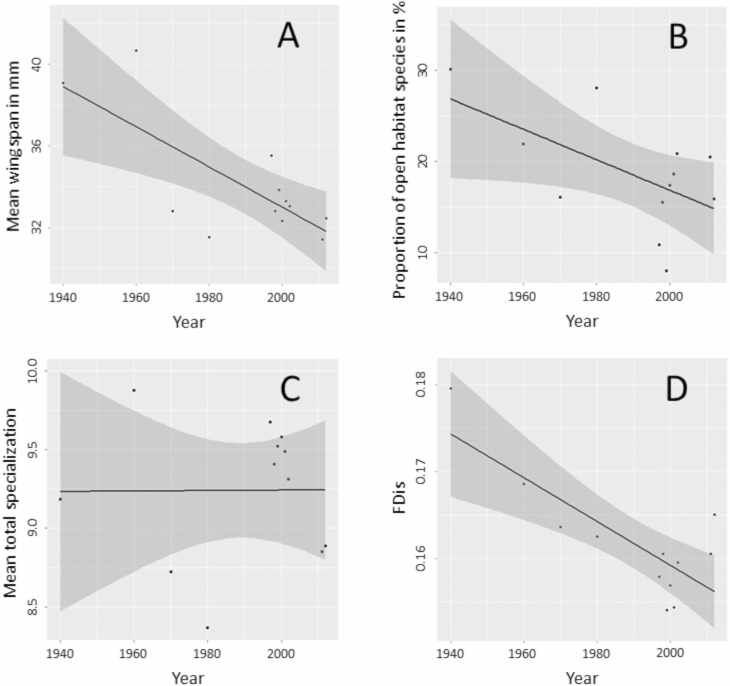
Plots showing (A) mean wingspan in millimeters, (B) proportion of open habitat species in percentage, (C) mean degree of total specialization, and (D) functional dispersion (FDis) in the moth community over time. Moth data from PsV were partitioned into 15 time layers for analysis. The dark line indicates the generalized additive model (GAM) function and its confidence intervals (shaded area). Statistical details of the GAMs can be looked up in Table 2.

In PsV, we counted 63 lost, 156 persistent, and 81 previously unrecorded moth species, summing up to a total of 300 species (Supp Table 1 [online only]). As predicted, locally lost species were on average larger than previously unrecorded moth species (*P* = 0.002, df = 287; Fig. 1, Table 2). The GAM also revealed substantial changes across the entire community in wingspan over time (Fig. 2A, Table 2).

**Table 2.** Results of generalized mixed-effect models for the three groups of ‘persistent’, ‘lost’, and ‘previously unrecorded’ moth species with regard to mean wingspan and ecological specialization score

	*t*-value/*z*-value	*P*-value	Regression coefficient β	Marginal *R*^2^	Conditional *R*^2^
1. Hypothesis Larger species have higher extinction risk					
Mean wingspan	**3.15**	**0.002**	**0.43**	**0.01**	**0.60**
2. Hypothesis Specialized species have higher extinction risk					
Total specialization	−1.22	0.22	−0.07	0.01	0.01
Food specialization	−0.26	0.79	−0.03	<0.01	<0.01
Habitat specialization	−0.76	0.45	−0.08	<0.01	<0.01
Northern distribution limit	−1.02	0.31	−0.09	<0.01	<0.01
Generalized additive models (observed change over 15 time periods)				Adjusted *R*^2^	
Mean wingspan	−**3.43**	**0.006**	−**0.73**	**0.5**	
Total specialization	0.02	0.98	0.01	−0.1	
Proportion of open habitat species	−1.90	0.09	−0.52	0.19	
Functional dispersion	−**4.16**	**0.002**	−**0.80**	**0.60**	

The results of the generalized additive models are also shown for mean wingspan, total specialization, and the proportion of open habitat species.

**Fig. 3.**
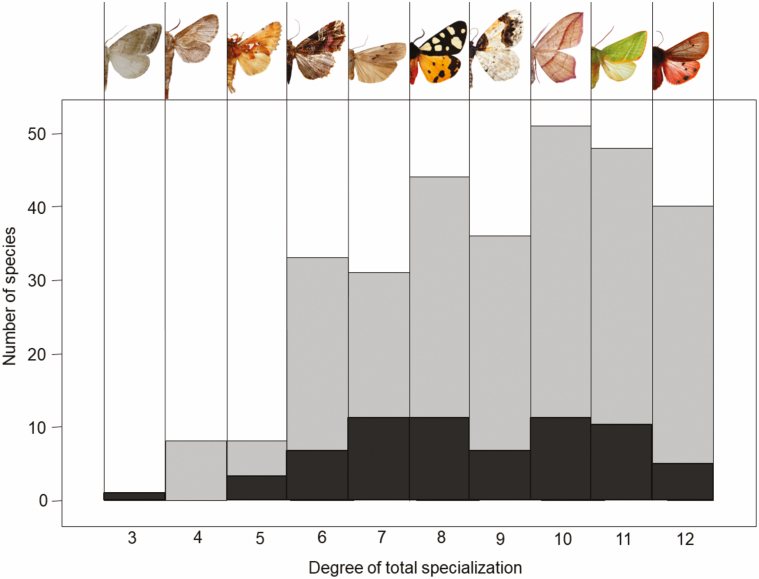
Number of species in regard to their total specialization score (gray bars). Black bars indicate the number of lost species. For each degree of specialization one representative species was selected viz. (from left): *Nola cristatula* Hübner 1783 (Lepidoptera: Nolidae), *Dyspessa ulula* Borkhausen 1790 (Lepidoptera: Cossidae), *Spatalia argentina* Schiffermüller 1775 (Lepidoptera: Notodontidae), *Callopistria juventina* Stoll 1782 (Lepidoptera: Noctuidae), *Arctia villica* Linnaeus 1758 (Lepidoptera: Erebidae), *Ligdia adustata* Schiffermüller 1775 (Lepidoptera: Geometridae), *Timandra comae* Schmidt 1931 (Lepidoptera: Geometridae), *Pseudoips prasinana* Linnaeus 1758 (Lepidoptera: Nolidae), *Phragmatobia fuliginosa* Linnaeuas 1758 (Lepidoptera: Erebidae).

Only one species, *Nola cristatula* Hübner, 1793 (Lepidoptera: Nolidae), reached the highest possible degree of specialization according to our classification system. Most moth species matched a specialization score of 10 or even higher, indicating a high proportion of generalist species in our data set (Fig. 3). Looking at the three groups of lost, persistent, and previously unrecorded species, the specialization scores showed no significant differences. This was true for all three dimensions separately—larval food, habitat, and northern distribution limit—as well as for total specialization (Table 2). Likewise, the GAM showed no consistent decline in total specialization or proportion of open habitat species over time (Figs. 2B and C). Contrary to these results, FDis declined significantly over the last 80 yr (*R*^2^ = 0.60, *P* = 0.002, Fig. 2D).

**Fig. 4.**
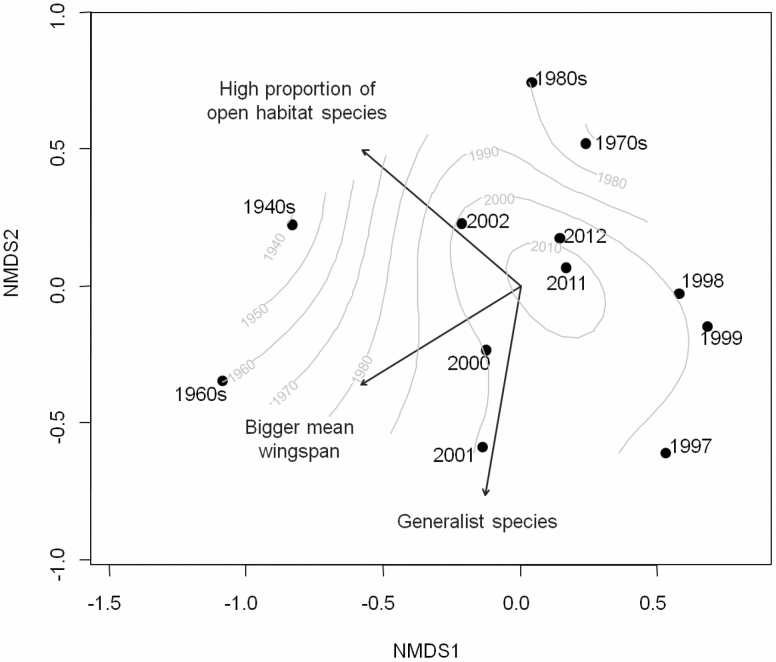
Non-metric multidimensional scaling (NMDS) ordination based on moth species lists from 15 time layers sampled in PsV. Proportion of open habitat species, mean wingspan, and total degree of specialization were superimposed as descriptors of the moth communities. The sampling years are projected on the NMDS through an ordisurf function based on a generalized additive model (GAM). Stress: 0.13 (non-metric fit *R*^2^ = 0.98, linear fit *R*^2^ = 0.91).

The unconstrained ordination plot reveals substantial changes in the species composition of moth assemblages over time (Fig. 4). According to a permutation test, variation in mean wingspan (*R*^2^ = 0.23, *P* = 0.001), mean total specialization (*R*^2^ = 0.19, *P* = 0.001), and the proportion of open habitat species (*R*^2^ = 0.14, *P* = 0.004) were all significantly associated with variation in species composition. However, moth samples partitioned into 15 time periods did not indicate a simple directional shift. Rather, older (museum) samples were ordinated in the periphery in reduced ordination space, with the most recent (quantitative light trap) samples in the center.

## Discussion

Even though they usually provide only snapshots of biota encountered at earlier times, museum collections may give important insight into past insect communities and therefore are valuable for the reconstruction of long-term community shifts. We were able to analyze changes in the PsV moth communities over the last 80 yr, where a clear shift in species composition has occurred. Larger species were more likely to go extinct locally, leading to a reduced community-wide mean wingspan over time. For ecological specialization, our results were contingent on the analytical method used. Although a coarse classification failed to reveal any differences in the degree of specialization between extinct and previously unrecorded species, a multivariate approach such as FDis well captured a significant decrease in specialization over the last 80 yr. Altogether the moth community changed significantly in PsV. These shifts in the moth assemblage seem to have been more intense in the first half of the 20th century because these oldest moth samples showed the largest differences to the recent ones, for example in the ordination analysis.

A higher rate of local losses among larger species was expected because similar developments have been observed with other insects in isolated conservation areas worldwide (Coulthard et al. 2019, Nolte et al. 2019). However, because our historic data were taken from museum vouchers rather than from standardized sampling or monitoring programs, one might ask to what extent apparent body size patterns might be shaped by the unknown whereabouts around the earlier samples. For example, citizen-scientists as collectors might have favored larger species or have overlooked small species. Dealing with historic data, there is always the problem that we have no information about how exactly the moths were sampled. We also do not know why a collector has taken, or discarded, particular species. For most historical natural history collections, personal interest in certain taxa or aiming to increase the completeness of the collection might have been decisive for the selection of the voucher individuals. Therefore, museum collections are obviously prone to sampling bias (Graham et al. 2004). So, proper adjustment of vouchers to be considered and a critical questioning of the results are crucial when historic collections are used for comparisons with more standardized surveys. In our case, however, such a sampling bias can mostly be refuted as many small and inconspicuous moth species were preserved in the evaluated historical collections (Wölfling et al. 2019). Furthermore, there are some obvious big moth species that have definitely disappeared from the area and have not been found again during our own intense sampling activities. For example, *Sphinx ligustri* Linnaeus, 1758 (Lepidoptera: Sphingidae), *Minucia lunaris* Denis & Schiffermüller, 1775 (Lepidoptera: Erebidae), *Catocala puerpera* Giorna, 1791 (Lepidoptera: Erebidae), or *Catocala elocata* Esper, 1787 (Lepidoptera: Erebidae) never showed up again after 1997, even though these moth species are easily recorded by light-trapping and are not considered as endangered in southern Europe. Hence, the fact that we never recorded any of these moths between 1997 and 2012 (and actually not beyond: B. Uhl and M. Wölfling, unpublished results) gives strong evidence that these large-sized species have really been lost in PsV.

During the observed time span, PsV did not suffer from any significant loss of area, nor did the host plants of these lost species disappear. We therefore assume that some other aspects of habitat quality or indirect effects of anthropogenic actions on the landscape scale might have been the determining factors that have led to the disappearance of these species.

Looking at the landscape scale, light pollution seems to have become more intense in recent years, caused by the development of the industrial harbor of Ravenna. Although Merckx et al. (2018) found that increasing urbanization favored bigger moth species, we observed an apparent selection against larger species. We can only hypothesize that this might be an effect of bigger species reacting differently to nearby light pollution sources. Mark and recapture experiments showed that moth families comprising large species, such as Sphingidae and Erebidae, tend to be attracted by light from wider distances than other families, e.g., Noctuidae or Geometridae (Merckx and Slade 2014). So, these families might be more affected by nearby light pollution around the reserve than others. However, the complex effects of light pollution on moths are only poorly understood to date (Owens et al. 2020), and so, the effect of light pollution on moth functional and physiological characteristics needs to be investigated in future analyses.

Besides the industrial development, agricultural intensification in the surroundings of PsV and accompanied nutrient influx has contributed to homogenization of the plant community in the park (Uhl et al. 2020). This may have filtered out some moth species with peculiar nutrient demands. Besides, elevated nutrient levels can alter food plant quality and directly loop back on the fitness of some moth species (Kurze et al. 2018). Finally, subsidence induced soil salinity (Antonellini and Mollema 2010) also might reduce food plant quality (Okon 2019). Elevated salt content in soil may influence the composition and structure of the mycorrhiza and can lead to the decline of mature trees (Montecchio et al. 2004). Massive damage on trees has already been observed all over PsV (Uhl and Wölfling 2015). As a bottom-up effect, salinity stress is known to influence the development and population parameters of insects (Quais et al. 2019). Whatever mechanistic pathway may have been involved, we therefore assume that larger species may have suffered more from the adverse effects of environmental stress, rendering them more prone to local extinctions.

For the degree in specialization, our results were strongly dependent on the method used to define specialization. Using only three coarse dimensions of ecological specialization (breadth of larval food niche, habitat preference, and northern distribution limit) yielded no clear patterns. In contrast, the result based on multivariate FDis was clearly significant. The small number of classification factors obviously resulted in a classification with too poor resolution. Furthermore, completely different ecological niches are rated with the same total score. For example, one only counts the numbers of used habitats or host plants, but does not distinguish between different types. Accordingly, the value of such simplified approaches is low, especially if alternative measures based on broader trait information is available. Fortunately, for European Lepidoptera, species–trait information is more complete than for most other insect orders (Ebert 1994‒2003).

**Fig. 5.**
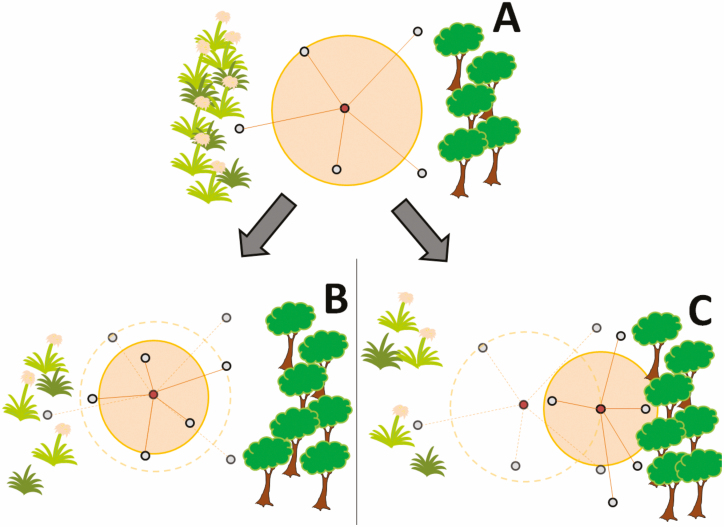
Change of functional dispersion over time in a conservation area where due to abandonment of land use and natural forest succession the open grassland biota gradually declines. Gray points represent species in functional trait space. The red point indicates the trait space center. The distance between each species and the trait space centroid is shown as a line. The orange circle symbolizes the mean functional dispersion FDis (mean distance across all species to the centroid). By simplifying multivariate trait space into two-dimensions, we indicate open habitat species to the left of the picture (grass symbols), and species of woody habitat on the right (tree symbols). (A) Situation about 80 yr ago with historic extensive land use. (B) First scenario: Species in general got ‘less specialized’. The functional centroid keeps its position, but individual species distances are on average smaller compared to the past (dashed lines). Consequently, mean functional dispersion decreases (compared with the ancient dispersion, shown as dashed circle). (C) Second scenario: Occupied functional trait space shifts toward more forest-affiliated species. Species might be also specialized, but predominantly concentrate in one habitat type. The dispersion of species toward open habitats therefore decreases, the trait space centroid shifts toward forest species, leading to smaller mean distances of species to the new trait space center.

For calculating FDis, the communities’ mean dispersion from the functional trait space centroid, a species–trait matrix consisting of multiple different ecological traits is used (Fig. 5A). Assuming that ‘specialization’ is the distance of each species from this centroid, a decline in FDis might indicate either a generally lower specialization of species in the community (Fig. 5B) or a shift of the moth community from multiple different niches to be occupied to one prevalent niche type (Fig. 5C). We know that in PsV the vegetation structure has changed during the last 80 yr due to natural succession (Wölfling et al. 2019). Following this process favoring more near-natural forest areas to develop, it is plausible that the moth community today is predominantly composed of forest-associated species. Functional dispersion of the community therefore would have shifted from species spread all over the trait space to species concentrated in the direction of forest dwellers (Fig. 5C). As we found no increase in strictly forest-bound species (Wölfling et al. 2019), we conclude that moderately generalistic species that depend on woody structures were responsible for the observed shift in FDis. In fact, the proportion of generalist habitat users has increased during the last 80 yr. With the proportion of open habitat species only slightly tending to decrease over time (Fig. 2B, Table 2), we furthermore conclude that within open habitats, there might have been a shift from strict open habitat users to those also tolerating or even preferring some bushy vegetation structures. Such a more subtle shift would have gone undetected with a coarse classification system. We therefore conclude that the observed change in community-wide FDis cannot be associated with one single habitat type or niche type that has vanished, but rather with multiple small changes in the occupied fraction of moth trait space.

Looking at the community composition over the last 80 yr, there was a significant change. Compositional differences between older samples were higher than among the newer ones, resulting in some kind of nested arrangement, with the earliest samples in the outer regions of the ordination plot, clearly distant from each other, and the newer own collections closer together in the center (Fig. 4).

For the analysis of species composition, the use of historic collections and different trap types has its limits. The collectors of the historic data likely did not sample in a standardized manner, but more likely have taken only individuals of interest. Vouchers of common species are more likely kept by entomologists only when a collection is started, while later on citizen-scientists may have focused on species missing in their collections. As a result, common species may not have been registered every year.

Using different light-trap types might have had an effect on the composition of the assemblages of attracted moths. We do not know which trap types were used by early collectors in Italy. Additionally, also the more recent samples are based on two different trap types (a manual light trap from 1997 to 2002 and in 2012; automated light traps in 2011). Different trap types are known to influence the composition of moth catches (Axmacher and Fiedler 2004). However, a large part of this influence is due to changes in relative abundances of individual species, and not so much in the presence or absence of species (Axmacher and Fiedler 2004). As we only used incidence data in our present analysis, and pooled all samples to one species list per year, we tried to minimize these sampling effects as best as possible.

Therefore, we suggest that the observed drastic alterations in species composition can also be attributed to the substantial environmental changes that have taken place in the reserve. When cattle were grazing under the pine trees, shrubs were regularly removed to facilitate pine-nut harvest, and fallen branches were collected as firewood. So, in these times, PsV still offered rather large tracts of open habitat due to frequent disturbances. More recently, the community seems to have become more stable, as community composition differences were smaller among the newer data sets. The recent forest-associated moth community of PsV can be seen as a consequence of secondary succession that started since the whole area received legal protection status in 1988. Also in other studies, moth communities of locations in mature forest systems were more similar than in secondary, younger forest types (Axmacher and Fiedler 2004).

This community similarity in recent times also suggests that the use of different types of traps (manual vs automated, each with different light sources) obviously did not have a major impact on the overall results of our study. Pooling the data by year therefore seems a useful procedure to make the incidence data from different trap types comparable.

Three key processes drive community assembly, viz. dispersal limitation, ecological filtering, and ecological drift (Sydenham et al. 2017). There is general evidence that larger moth species disperse more easily (Ockinger et al. 2010, Kuussaari et al. 2014). But for most of the species, there is a lack of knowledge concerning their dispersal capacity. So, it is quite difficult to make any assumptions about this important point driving community assembly. However, PsV—although being isolated from other forest areas—is still connected with other near-natural areas like wetlands and open habitats in the North. Furthermore, there are conservation efforts, trying to better connect PsV with other reserves (Estreguil et al. 2013). Streets are often edged by bigger trees, hedgerows, and field edges with various grass and herb species, and individual trees can often be found in agricultural areas around PsV. Such small microhabitats may serve as important stepping stones, facilitating dispersion between natural areas (Slade et al. 2013). As such, dispersal limitation should not be the major threat to the PsV moth communities, as is also indicated by the rather large number of previously unrecorded species over time.

With regard to ecological filters, we conclude that the abandonment of extensive use of forest commodities after the 1970s and subsequent succession were the major drivers of the observed moth community shift. This led to a reduction in the proportion of open habitat species and a shift toward more generalist forest species. However, the loss of open areas was recognized in time, so that conservation efforts in PsV were attempted to keep the structurally rich habitats. Therefore, horses were released as ecosystem engineers, keeping open areas free from bushes and counteracting natural succession.

Concerning ecological drift, it is often overlooked that local populations may go extinct due to stochastic reasons even in the absence of environmental stress, such that changes in community composition can be mistaken for indicating an environmental trend (Sgardeli et al. 2016). Therefore, some random extinctions may have occurred. For example, also small and rather generalistic species such as *Eupithecia absinthiata* Clerck, 1759 (Lepidoptera: Geometridae) and *Tephronia sepiaria* Hufnagel, 1767 (Lepidoptera: Geometridae) appear to have disappeared. Due to our intensive sampling, we are confident that *E. absinthiata* was likely not overlooked. *Tephronia sepiaria* has disappeared although other lichen feeders are still found in numbers in PsV. Simultaneously, large species such as *Mormo maura* Linnaeus, 1758 (Lepidoptera: Noctuidae) and *Deilephila porcellus* Linnaeus, 1758 (Lepidoptera: Sphingidae) were previously unrecorded. The latter two species would definitely have been documented by earlier collectors, had they ever encountered them.

In summary, the moth communities in PsV currently seem mostly to be shaped by ecological filters combined with random ecological drift. With the protection status conferred to the area in the late 1980s, most constraints driven by former extensive forest use were suspended. For the preservation of open habitat structures in PsV, nowadays horses are held within the reserve. By doing so, the diversity of different habitat structures is maintained. Additionally, with a relatively large area of about 900 ha, PsV seems to be able to preserve also a range of specialized insect species (Slade et al. 2013).

## Supplementary Material

ieaa097_suppl_Supplementary_Appendix_1
